# Differential requirements for *Gli2* and *Gli3* in the regional specification of the mouse hypothalamus

**DOI:** 10.3389/fnana.2015.00034

**Published:** 2015-03-25

**Authors:** Roberta Haddad-Tóvolli, Fabian A. Paul, Yuanfeng Zhang, Xunlei Zhou, Thomas Theil, Luis Puelles, Sandra Blaess, Gonzalo Alvarez-Bolado

**Affiliations:** ^1^Department of Medical Cell Biology and Neuroanatomy, University of HeidelbergHeidelberg, Germany; ^2^Laboratory of Neurodevelopmental Genetics, Institute of Reconstructive Neurobiology, Life and Brain Center, University of BonnBonn, Germany; ^3^Centre for Integrative Physiology, University of EdinburghEdinburgh, UK; ^4^Department of Morphology, Instituto Murciano de Investigación Biosanitaria, School of Medicine, University of MurciaMurcia, Spain; ^5^Facultad de Medicina, University of MurciaMurcia, Spain

**Keywords:** embryo, *Gli1*, *Gli2*, *Gli3*, hypothalamus, mouse, mutant, Shh

## Abstract

Secreted protein Sonic hedgehog (Shh) ventralizes the neural tube by modulating the crucial balance between activating and repressing functions (GliA, GliR) of transcription factors *Gli2* and *Gli3*. This balance—the Shh-Gli code—is species- and context-dependent and has been elucidated for the mouse spinal cord. The hypothalamus, a forebrain region regulating vital functions like homeostasis and hormone secretion, shows dynamic and intricate *Shh* expression as well as complex regional differentiation. Here we asked if particular combinations of *Gli2* and *Gli3* and of GliA and GliR functions contribute to the variety of hypothalamic regions, i.e., we wanted to approach the question of a possible hypothalamic version of the Shh-Gli code. Based on mouse mutant analysis, we show that: (1) hypothalamic regional heterogeneity is based in part on differentially stringent requirements for *Gli2* or *Gli3*; (2) another source of diversity are differential requirements for Shh of neural vs. non-neural origin; (3) the medial progenitor domain known to depend on *Gli2* for its development generates several essential hypothalamic nuclei plus the pituitary and median eminence; (4) the suppression of Gli3R by neural and non-neural Shh is essential for hypothalamic specification. Finally, we have mapped our results on a recent model which considers the hypothalamus as a transverse region with alar and basal portions. Our data confirm the model and are explained by it.

## Introduction

Sonic hedgehog (Shh) is a morphogen required for ventral neural tube specification (Echelard et al., 1993; Ericson et al., 1995, 1997; Chiang et al., 1996). Shh acts through the Gli transcriptional activators (GliAs) and repressors (GliRs); the balance between GliA and GliR specifies ventral differentiation and proliferation (Lee et al., 1997; Ruiz i Altaba, 1997; Brewster et al., 1998). This “Shh-Gli code” is known for the mouse spinal cord [reviewed in Ruiz i Altaba et al. (2003), Dessaud et al. (2008), Ingham et al. (2011)] and brainstem (Wang et al., 1995; Blaess et al., 2006, 2011; Feijoo et al., 2011).

The Shh expression domain in the forebrain is more extensive and elaborate than in the spinal cord, has become more intricate and dynamic during phylogenesis and is considered a motor of brain evolution (Osorio et al., 2005). Work on thalamic development supports the notion that regional variation of the Shh-Gli code underlies forebrain complexity (Haddad-Tovolli et al., 2012). In the same way, the canonical Shh-Gli code shows interspecies variation (Ruiz i Altaba, 1997; Aberger and Ruiz, 2014). On the basis of mutant phenotype analysis at different rostro-caudal levels of the mouse spinal cord and the hindbrain it has been proposed that *Gli2* and *Gli3* have partially overlapping roles and that their relative contributions to ventral specification shows regional variation (Motoyama et al., 2003; Lebel et al., 2007).

**FIGURE 1:**
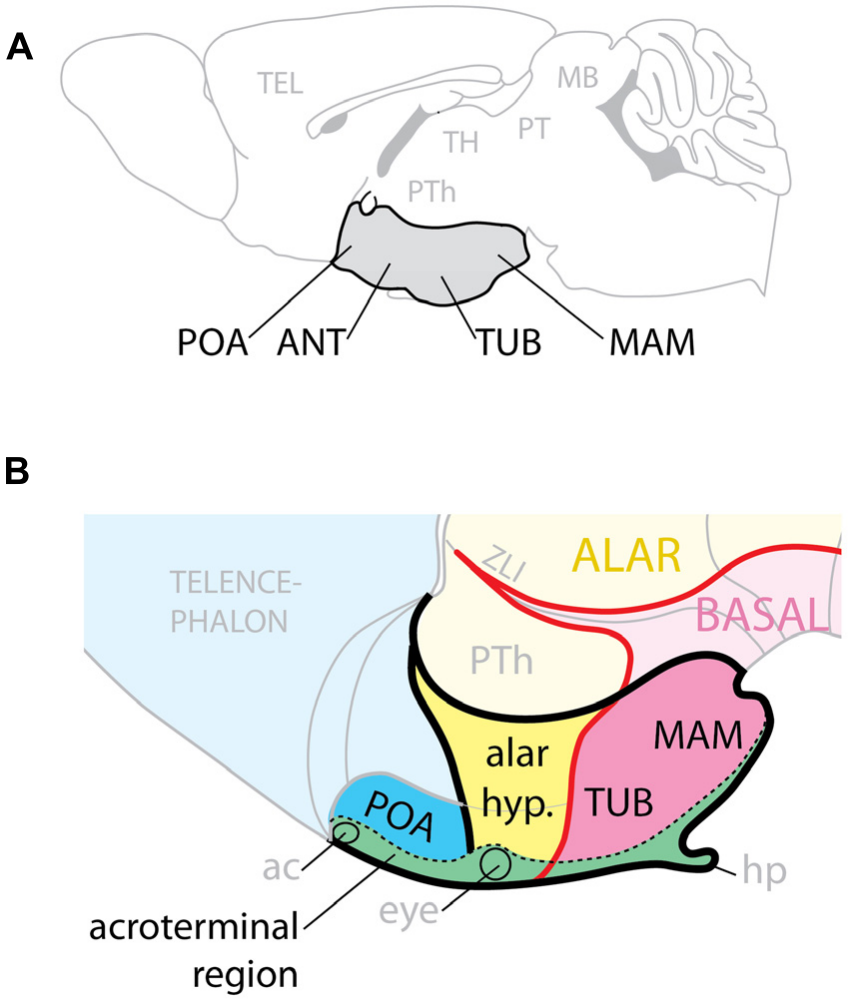
**Hypothalamic regions. (A)** Conventional representation of the hypothalamus (gray) as ventral region with four rostro-caudal regions, POA, preoptic; ANT, anterior; TUB, tuberal; MAM, mamillary. **(B)** Model of the hypothalamus considering Shh expression (pink) as basal (ventral) marker. The POA is part of the telencephalon; the alar hypothalamus (yellow) corresponds to the anterior region; the tuberal and mamillary regions are not “caudal” but basal (ventral). ac, anterior commissure; hp, hypophysis; PTh, prethalamus; ZLI, zona limitans.

The hypothalamus regulates homeostasis, endocrine secretion, and reproductive behavior (Saper, 2006; Puelles et al., 2012; Sternson, 2013) and its alterations can cause conditions like obesity and high blood pressure (Caqueret et al., 2005; McMillen et al., 2008). Complex gene expression pattern combinations underlie hypothalamic regional specification (Shimogori et al., 2010; Puelles et al., 2012). On the basis of classical neuroanatomy studies, the adult hypothalamus has been traditionally described as subdivided into four regions (preoptic, anterior, tuberal, and mamillary) arranged rostro-caudally and ventrally in the brain (**Figure 1A**) and flanked by the lateral hypothalamic area (LHA), a large region essential to regulate behavioral state and arousal (Swanson, 1987). The modern view considers the adult hypothalamus as part of a behavioral control column (Swanson, 2000).

Here we analyze the hypothalamic phenotypes of mouse mutants to ascertain which combinations of GliA and GliR specify the mouse hypothalamic regions and which Gli genes perform these functions. We examined embryos primarily after mid-gestation so that we could assess which hypothalamic nuclei are affected when the GliA/R code is affected. We have mapped our results on a model of the developing hypothalamus (Puelles et al., 2012; **Figure 1B**) built around the observation that, since *Shh* is indispensable to ventralize the neural tube, and it is expressed in a long domain stretching the entire length of it, it follows that during development the *Shh* expression boundary separates dorsal (alar) from ventral (basal). The rostral end of the developing neural tube is closed by a transverse structure called acroterminal region, which does not share the typical characteristics of the floor plate. Beyond mamillary level, the acroterminal region extends all the way through the tuberal region, alar hypothalamus and preoptic region and up to the anterior commissure, it is transversally oriented (has alar and basal portions) and strongly patterned (probably by the underlying prechordal plate) and would generate the median eminence, infundibulum, neurophypophysis, preoptic terminal lamina, eyes, optic chiasma, and suprachiasmatic area. Two progenitor domains, medial and lateral, give rise to the basal part of the hypothalamus: the medial domain generates median eminence and neurohypophysis, medial portions of the ventromedial and arcuate nuclei, and the mamillary body; the lateral originates most of the ventromedial nucleus, the dorsomedial nucleus and the LHA (Alvarez-Bolado et al., 2012).

We show that, in the basal hypothalamus, the medial progenitor domain requires non-neural Shh acting through Gli2A. The lateral progenitor domain is patterned by neural Shh acting through Gli3R and Gli2A or Gli3A. In the presence of Shh signaling, the Gli3R function is not required for hypothalamus specification. Neither *Gli2* nor *Gli3* are required for overall patterning of the alar hypothalamus and preoptic area. These data confirm the main tenets of the model (Puelles et al., 2012), since they strongly support a subdivision of the developing hypothalamus into alar and basal domains.

## Materials and Methods

### Mice and Mouse Lines

Animals were housed and handled in ways that minimize pain and discomfort, in accordance with German animal welfare regulations (TierSchG) and in agreement with the European Communities Council Directive (2010/63/EU). The authorization for the experiments was granted by the Regierungspräsidium Karlsruhe (state authorities) and the experiments were performed under surveillance of the Animal Welfare Officer responsible for the Institute of Anatomy and Cell Biology. To obtain embryos, timed-pregnant females were sacrificed by cervical dislocation; the embryos were decapitated.

#### *Gli2*^**zfd/+**^ (*Gli2* Zinc Finger-Deleted) Mutant Mice

This *Gli2* null mutant mouse line was generated (Mo et al., 1997) by replacing the exons encoding for zinc fingers 3–5. The deletion leads to an out-of-frame mutation causing disrupted transcription from the deletion site to the 3 end of the *Gli2* gene. This results in translation of a truncated protein unable to bind to DNA, since the zinc fingers 4 and 5 are essential for DNA binding (Pavletich and Pabo, 1993). The *Gli2^zfd/zdf^* are null mutants for *Gli2*; the *Gli2^zfd/+^* have normal phenotypes and are used as controls.

#### *Gli3^**Xt-J/+**^* (Extra-Toes) Mutant Mice

This line carries a 50 kb deletion that removes the exons encoding zinc fingers 3–5 and the complete 3^′^ part of the *Gli3* gene (Hui and Joyner, 1993; Maynard et al., 2002; Genestine et al., 2007). The *Gli3^Xt-J/Xt-J^* are null mutants for *Gli3*.

We have not been able to obtain double *Gli2-Gli3* mutant embryos (*n* = 4 litters, 1 at E9.5, 2 at E10.5, and 1 at E12.5).

#### *Gli3-nlacZ* Mutant Mice

The *Gli3-nlacZ* knock-in mouse line was generated by partially replacing the first coding exon of *Gli3* with the nlacZ cDNA. Thus, expression of lacZ is controlled by the endogenous *Gli3* promoter/enhancer elements and can be used to monitor the expression pattern of *Gli3* (Garcia et al., 2010).

#### *Foxb1-Cre* Mutant Mice

Express Cre in the thalamic and hypothalamic neuroepithelium (Zhao et al., 2007, 2008). We used only heterozygous* Foxb1-Cre* mice, which show a normal phenotype (Zhao et al., 2007, 2008), *Foxb1 Cre/Cre* homozygotes were not used in this study.

#### *Foxb1-Cre;Shh^**f/+**^* Mutant Mice

To obtain mice specifically deficient in Shh expressed in the neural tube (conditional knock-out for neural* Shh)*, we crossed our *Foxb1-Cre* mice (Zhao et al., 2007, 2008) with *Shh^f/+^* conditional mutants in which exon 2 of the *Shh* gene was flanked by loxP sites (Dassule et al., 2000; Lewis et al., 2001). The *Shh^f/+^* conditional mutants were generated in the laboratory of Dr. Andrew McMahon (University of Harvard) and were obtained through Jackson Labs (www.jax.org). The *Foxb1-Cre;Shh^f/f^* mutants lack all Shh expression in the forebrain neuroepithelium (Szabo et al., 2009a,b).

#### *Foxb1-Cre;Shh^**f/+**^;Gli3^**Xt-J/+**^* Mutant Mice

The double homozygous mutants for *Shh* expressed in the neural tube (neural *Shh* or n*Shh*) and *Gli3* were generated by crossings between *Foxb1Cre;Shh^f/+^* mice; and *Gli3^Xt-J/+^* mice. The double mutants (*Foxb1-Cre;Shh^f/f^;Gli3^Xt-J/^^Xt-J^*) do not survive beyond birth.

### *In Situ* Hybridization

Embryos or embryonic brains were dissected, fixed in 4% paraformaldehyde, and embedded in paraffin. Non-radioactive ISH was performed on paraffin sections (7 μm for E10.5, 10 μm for E12.5, and 14 μm for E18.5 embryos) that were fixed in 4% paraformaldehyde and acetylated after sectioning. RNA *in situ* hybridization was performed as described (Blaess et al., 2011).

### BrdU Labeling

Pregnant mice (E12.5) from appropriate crossings were injected intraperitoneally with 5^′^-bromo-2^′^-deoxyuridine (BrdU; Sigma; 50 μg/g body weight) at 12:00 h. Three hours after the injection, embryos were collected and fixed overnight in 4% PFA in PBS at 4°C. Cell proliferation was detected by means of antibody (rat anti-BrdU; AbCam; 1:100) after epitope retrival in Tris-EDTA buffer pH = 9.0 for 20 min in pressure cooker. The nuclear marker 4^′^6-diamidino-2-phenylindole dihydrochloride (DAPI; Invitrogen) was used as a counterstain. For cell counting, 10 μm paraffin sections were analyzed under a confocal microscope (LSM700 -Zeiss) and DAPI and BrdU-positive cells were counted in 100-μm-wide bins encompassing the thickness of the neuroepithelium (apical to basal side) at four hypothalamic sites (preoptic area, alar hypothalamus and tuberal and mamillary regions) on two histological sections per level in three animals per age and genotype (WT, *Gli2^zfd/zfd^*, *Gli3^Xt-J/Xt-J^*, and *Foxb1-Cre;Shh^f/+^;Gli3^Xt-J/+^* double mutants). The BrdU-labeling index (BrdU-labeled cells as percentage of total cells) was then calculated (Takahashi et al., 1993; Warren et al., 1999; Ishibashi and McMahon, 2002).

### Cloning of Constructs

In an expression vector driven by pCAGGS (Niwa et al., 1991) we inserted either EmGFP (kind gift of Dr. Boris Fehse, University of Hamburg; Weber et al., 2010) or tdTomato (kind gift of Dr. Roger Y. Tsien, UCSD) as reporters. On vectors carrying the tdTomato reporter we then inserted (upstream an internal ribosomal entry site and the reporter) a mutated form of human PTCH1 in which we deleted part (between MfeI and NsiI) of the second large extracellular loop (PTCH1-Δ-loop2), as was done in Briscoe et al. (2001).

### *In Utero* Electroporation

This procedure was carried out as described (Saito and Nakatsuji, 2001; Saito, 2006; Haddad-Tovolli et al., 2012) with added caveats for hypothalamus targeting (Haddad-Tovolli et al., 2013). Pregnant mice at E12.5 were anesthetized with a mixture of Halothane (Isoflurane, Baxter) and oxygen (0.5 l/min) administered with a Komesaroff Anaesthetic Machine. The uterus was exposed and the DNA solution (1 μg/μl) was injected with a glass micropipette in the third ventricle of the embryo brain through the uterine wall. Electric pulses were administered with a CUY21 electroporator (Nepagene, Japan; 5 square-wave pulses, 50 V, 50 ms on/950 ms off) and a stainless steel needle electrode (CUY550-10) used as positive pole and a round flat electrode (CUY700P4L) as negative pole. After the surgery, the embryos were allowed to develop *in utero* for 6 days and collected at E18.5 for analysis. The embryonic brains were dissected, fixed overnight in 4% PFA in PBS at 4°C and then protected with sucrose (20; 30%) and embedded in OCT mounting medium (Tissue Tek). Blocks were sectioned into 20 μm thick sections in a cryostat (Leica CM3050S) and observed and photographed with a Zeiss LSM 700 confocal microscope. We used laser line 488 nm for the green reporter EmGFP (excitation maximum 487 nm, emission maximum 509 nm) and laser line 555 nm for the red reporter tdTomato (excitation maximum 554, emission maximum 581). Since our readouts are based in the comparison between numbers of cells counted on confocal images (see next paragraph), it was imperative to relie on strictly comparable data. To guarantee comparability, the images of experimental and control brains were obtained under the exact same conditions and with the exact same confocal settings.

### Experimental Design of *In Utero* Electoporation Experiments

Because each *in utero* electroporation experiment results in a different number of neuroepithelial cells being transfected, the experiments are not directly comparable with each other. For this reason we do “two-reporter-experiments” (Haddad-Tovolli et al., 2012). The two reporters answer two problems. The green reporter construct (GFP) is an internal control. It will label every one of the transfected neuroepithelial cells and their progeny. In this way, for each single electroporated mouse embryo we know how many cells have been transfected. The second question is the actual scientific question: “does *Ptch1-delta-loop2* reduce proliferation?” For this, we have a second construct expressing a dominant loss-of-function version of the Ptch1 receptor (Ptch1-delta-loop2, see above Cloning of constructs) and, in the same construct, a red reporter (tandem dimer tomato, tdTomato). We use a ratio of 2 (GFP):1 (Ptch1-delta-loop2+red) in order to introduce some bias in the results, so that the readout of the experiment is the ratio between green cells and red cells. In principle there must be, after electroporation, a very few cells which are only green: they happen to express only GFP (not Ptch1-delta-loop2+red), proliferate normally and generate numerous green neurons, otherwise presumably normal. If the cells coexpressing the green plus the red (= experimental) constructs proliferate less, we will see less green + red neurons.

In parallel, we performed control experiments transfecting a 2:1 mixture of GFP construct and tdTomato construct (without loss-of-function Ptch1 protein) in order to evaluate how many only green and how many green-plus-red neurons we obtain in normal circumstances (i.e., without introducing any dominant loss-of-function). Those are the gray bars in **Figure 10J**. Additionally, these control experiments remove a possible concern related to the relative brightness of the green and the red reporters. In principle, a green cell could have been transfected also with some red (experimental) constructs in a number to small to be detected (since EmGFP is brighter than tdTomato). This possible source of imprecision can be disregarded since our readout is not absolute but relative (comparison between gray bars and black bars; **Figure 10J**).

### Statistics

Statistical assessment of the BrdU and electroporation data was performed with Prism 6 software (Graph Pad Software, San Diego, CA, USA).

### Morphological Interpretive Model

The results of mutant analysis were interpreted and mapped using the updated prosomeric model (Puelles et al., 2012) and the Allen Brain Atlas (Allen-Institute-for-Brain-Science, 2009).

## Results

### Developing Hypothalamic Expression of *Shh* and *Gli* can be Broadly Subdivided into at least Three Stages

**FIGURE 2:**
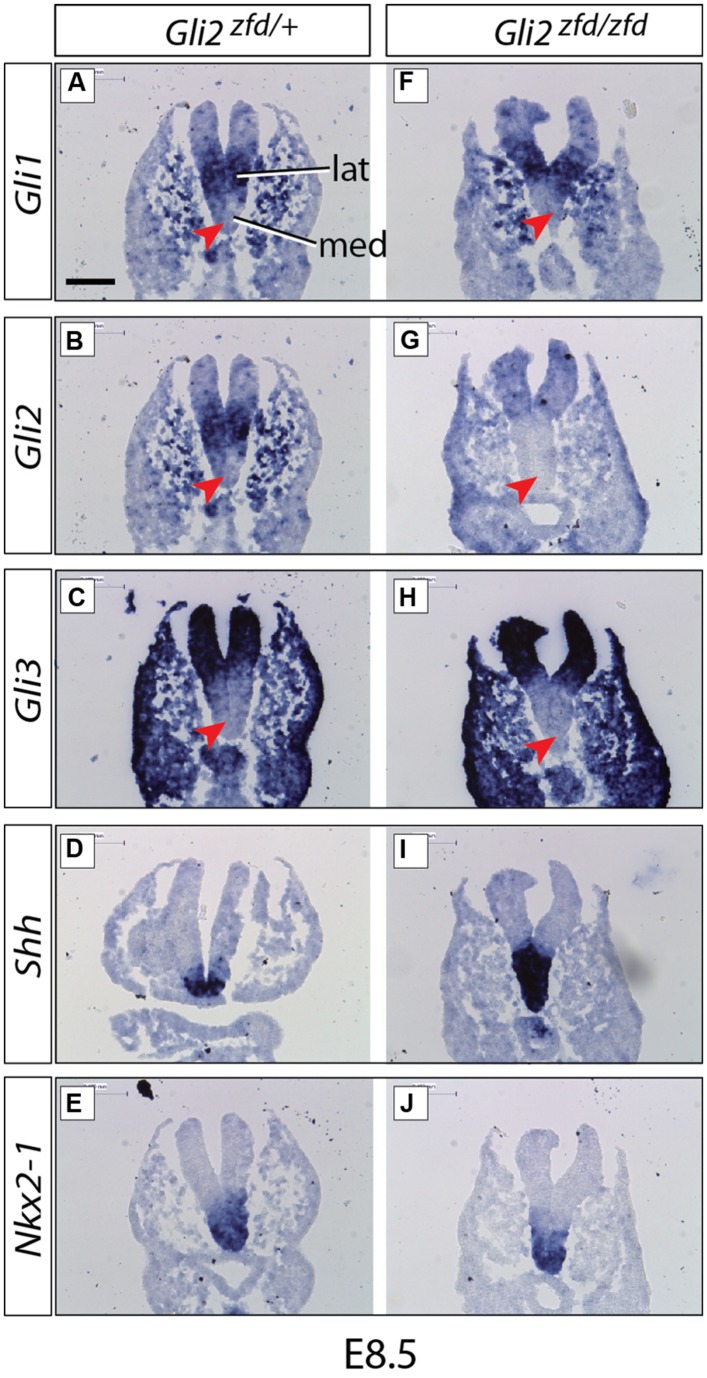
**Expression of *Gli* genes in the presumptive hypothalamus at E8.5.** *In situ* detection of marker gene expression in *Gli2^zfd/+^* and *Gli2^zfd/zfd^* mutant E8.5 embryos as indicated. “lat” and “med” in **(A)** indicate progenitor domains. Red arrowheads in **(A–C,F–H)** indicate lack of expression in the medial progenitor domain. *Nkx2-1* expression **(E,J)** identifies the presumptive hypothalamus. Scale bar **(A)** 100 μm.

**FIGURE 3:**
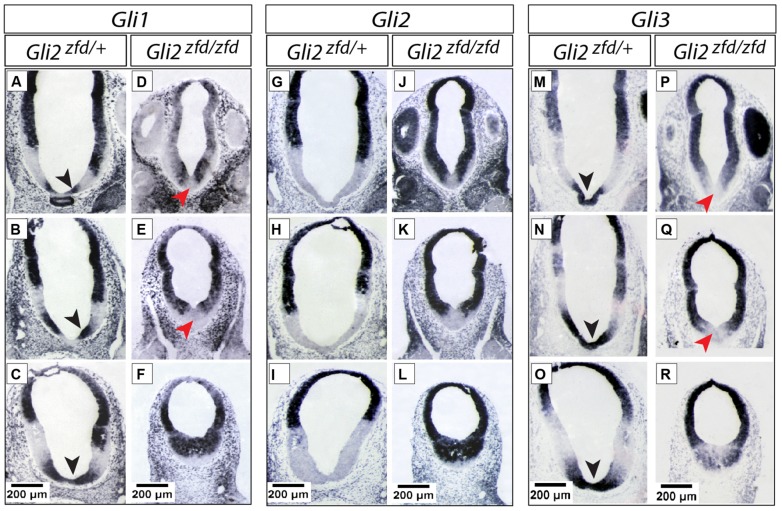
**Expression of *Gli* genes in the presumptive hypothalamus at E10.5. (A–R)** *In situ* detection of *Gli* genes in the presumptive hypothalamus of E10.5 *Gli2^zfd/+^* and *Gli2^zfd/zfd^* mouse embryos as indicated. For each gene, three levels are shown, from pituitary/infundibulum (top row) through mamillary region (bottom row). Black arrowheads in **(A–C,M–O)** show co-expression of *Gli1* and *Gli*3 in the midline; red arrowheads in **(D,E,P,Q)** show downregulation of *Gli1* and *Gli3* in the *Gli2^zfd/zfd^* midline. Scale bars, 200 μm.

Our purpose was to determine for each of the mouse hypothalamic regions which member of the Gli family performs the GliA and which one the GliR function, and which combinations of GliA and GliR specify these regions—in short, the hypothalamic Shh-Gli code. The expression of *Gli1, Gli3,* and Shh has been assessed at several stages in the developing chick hypothalamus, but in mouse the data are less comprehensive (Aoto et al., 2002; Ohyama et al., 2008). Thus, the first requisite for our study was to ascertain a detailed spatial-temporal expression map for the three mammalian *Gli* genes and *Shh* in the developing hypothalamus of the mouse (**Figures 2** and **3**). Although inactivation of *Gli1* does not result in an abnormal phenotype (Park et al., 2000; Bai et al., 2002), *Gli1* expression is a readout for Shh signaling [see references in Lewis et al. (2001)] and for this reason it was important to analyze its expression domain too. It has been described that, in the mouse neural plate, expression of *Gli* genes is first detected at E7.5 (neural fold); in this early stage of *Gli* expression, *Gli1* is expressed only in the midline of the neural fold, while *Gli2* and *Gli3* expression is widespread in the entire ectoderm (Hui et al., 1994) and *Shh* is expressed in the underlying mesoderm (non-neural Shh; Echelard et al., 1993).

We started our investigation of *Gli* expression patterns after neurulation, when they become more complex and at the same time more relevant to our study. At E8.5 (middle stage; **Figure 2**), *Gli1* and *Gli2* were expressed in overlapping patterns in the lateral domain (Alvarez-Bolado et al., 2012; **Figures 2A,B**), while *Gli3* was expressed in a more peripheral, non-hypothalamic domain (**Figure 2C**) and *Shh* was expressed in the medial domain (neural *Shh,* medial expression; **Figure 2D**), in coincidence with the medial progenitor domain (Alvarez-Bolado et al., 2012). The presumptive hypothalamus was defined by expression of specific marker *Nkx2-1* (**Figures 2E,J**).

At E10.5 (late stage; **Figure 3**) the Gli expression pattern had changed again. While *Gli2* expression was absent from the hypothalamic primordium (**Figures 3G–I**), *Gli3* and Shh-activation diagnostic marker *Gli1* showed overlapping expression domains in the medial domain (**Figures 3A–C,M–O**), suggesting a potential activator function of *Gli3* (Gli3A) in the midline at this age. *Shh* was expressed in a lateral domain corresponding to the lateral progenitor domain (Alvarez-Bolado et al., 2012; neural *Shh*, lateral expression). We concluded that the hypothalamic expression of *Shh* and the *Gli* genes can be broadly subdivided into at least three stages (summarized in **Figure 11A**).

### Deficiency in *Gli2* or *Gli3* does not Alter the Overall Specification of the Alar Hypothalamus

**FIGURE 4:**
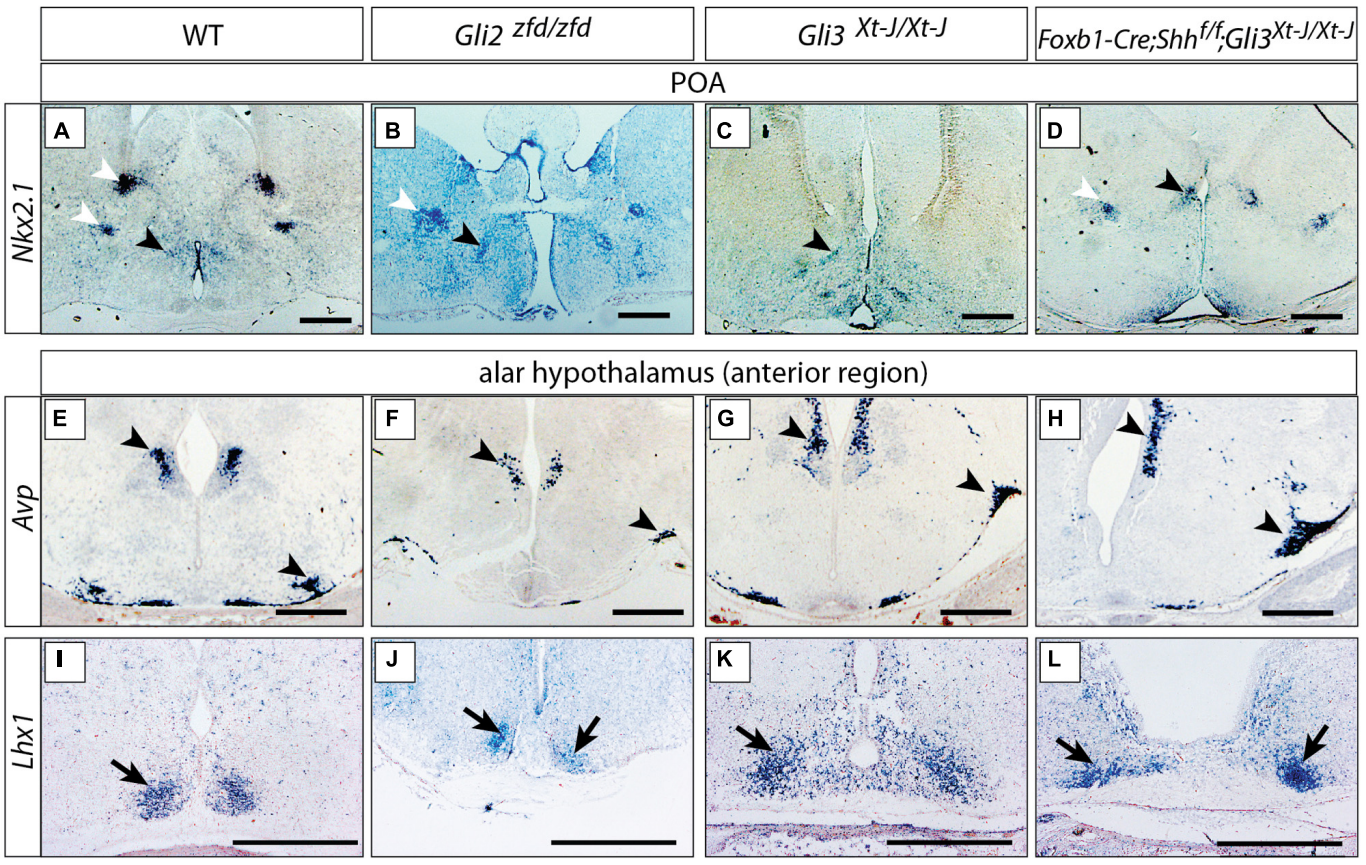
**Either Gli2A or Gli3A is sufficient for the specification of the preoptic region and alar hypothalamus. (A–D)** *In situ* detection of preoptic marker gene *Nkx2.1* on E18.5 mouse brain sections, genotypes as indicated. Black arrowheads, preoptic neuronal nuclei; white arrowheads, additional telencephalic expression domains. **(E–L)** *In situ* detection of anterior marker genes on E18.5 mouse brain sections, markers and genotypes as indicated. Arrowheads in **E–H** indicate the supraoptic and paraventricular nuclei; arrows in **I–L** indicate the suprachiasmatic nucleus. Scale bars 500 μm.

Sonic hedgehog is required to specify hypothalamic structures and the preoptic area (Chiang et al., 1996; Pabst et al., 2000; Rallu et al., 2002). In mouse mutants lacking *Shh* expression in the neural tube (*Foxb1-Cre;Shh^f/f^* mutants), however, the preoptic and alar hypothalamus have only a moderate phenotype, mostly evident in their reduced size (Szabo et al., 2009a; Zhao et al., 2012), indicating that they are specified by Shh of non-neural origin (e.g., from the prechordal plate or the notochord). Here we asked what is the role of Gli factors in those two hypothalamic regions by analyzing mutants in which *Gli2, Gli3* or both neural *Shh* and *Gli3* were inactivated. Expression of transcription factor *Nkx2-1,* an early preoptic marker (Shimamura et al., 1995; Xu et al., 2008), was preserved in the *Gli2^zfd/zfd^* and *Gli3^Xt-J/Xt-J^* mutants (black arrowheads in **Figures 4A–C**).Incidentally, some non-preoptic telencephalic expression domains were missing in the mutants (white arrowheads in **Figures 4A–D**). *Arginin-Vasopressin (Avp)* is specifically expressed by the supraoptic and paraventricular nuclei (Swanson and Sawchenko, 1983) and shows robust expression in both mutants (**Figures 4E–G**). The transcription factor gene *Lhx1* is a marker of the suprachiasmatic nucleus (Szabo et al., 2009a), and this pattern remains essentially unchanged in the mutants (**Figures 4I–K**). Finally, analysis of double *Foxb1-Cre;Shh^f/f^;Gli3^Xt-J/Xt-J^* mutants (lacking both neural *Shh* and *Gli3*) showed robust marker expression (**Figures 4D,H,L**).

These results could indicate that, downstream of Shh of non-neural origin at the early stage, *Gli2* and *Gli3* can fully substitute for each other’s activator function in the alar portions of the hypothalamus or, alternatively, that the specification of the alar hypothalamus depends on suppression of Gli3R by non-neural *Shh* (Rallu et al., 2002).

### *Gli2* is Required for the Development of Medial Tuberal and Mamillary Regions

**FIGURE 5:**
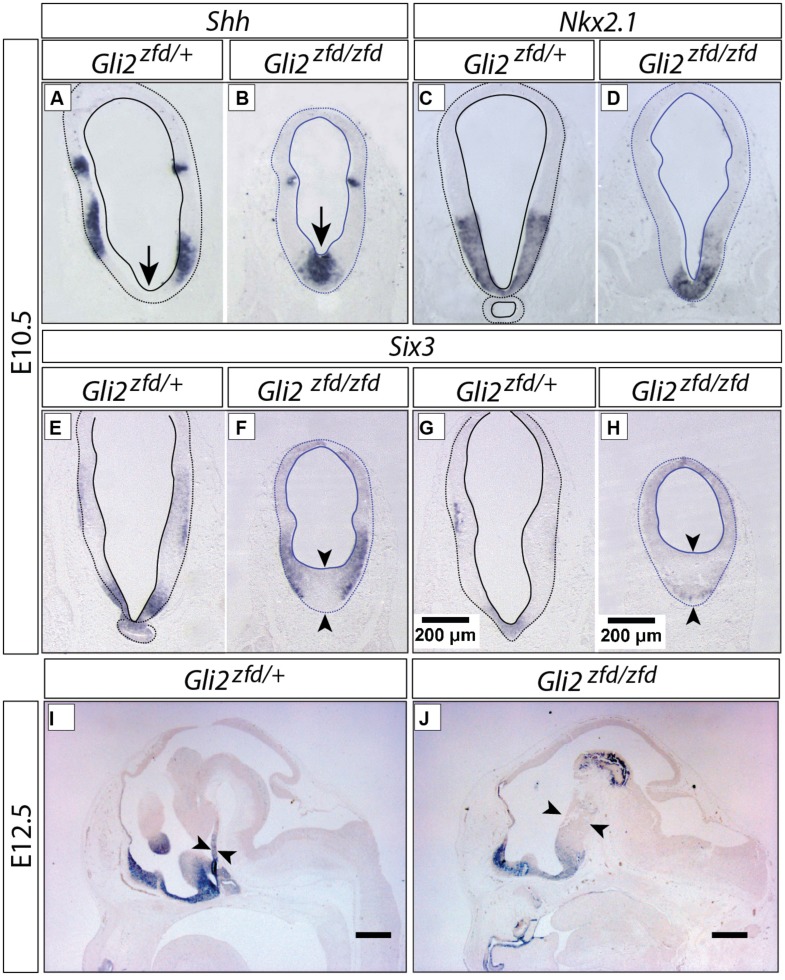
**Abnormal medial domain in the *Gli2^zfd/zfd^* mutant. (A–H)** *In situ* detection of gene expression on forebrain sections of E10.5 embryos, genotypes, and markers as indicated. In **(E–H)**, pituitary **(E,G)** and median eminence **(F,H)** levels are shown. Scale bar in **(G,H)**, 200 μm. **(I,J)** *In situ* detection of *Six3* expression on sagittal sections of E12.5 WT **(A)** and *Gli2^zfd/zfd^* **(B)** mouse embryos. Arrows show thickness of midline. Scale bars, 500 μm.

**FIGURE 6:**
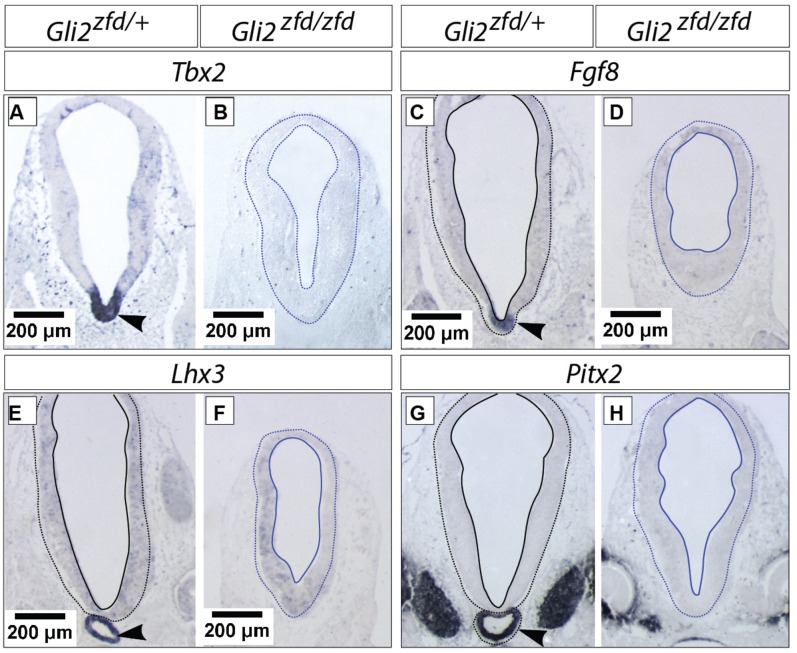
**Hypophysis region in the *Gli2^zfd/zfd^* mutant at E10.5.** *In situ* detection of infundibular and pituitary markers on *Gli2^zfd/+^* and *Gli2^zfd/zfd^* E10.5 embryos as indicated. Arrowheads in **(A,C,E,G)** indicate normal expression domain. Scale bars, 200 μm.

In order to analyze the *Gli2^zfd/zfd^* phenotype in the basal hypothalamus (tuberal and mamillary regions), we examined expression of *Shh* and *Gli* genes as well as regional markers at E8.5 and E10.5. At E8.5, expression of *Gli1*, *Gli3* and the regional marker *Nkx2-1* was not changed in the *Gli2^zfd/zfd^* mutant (**Figures 2F,H–J**), except of course for the disappearance of the *Gli2* domain (**Figure 2G**). Expression of Shh, however, seemed expanded (**Figure 2I**). At E10.5, expression of *Gli1* and *Gli3* was strongly downregulated in the midline around the infundibular area (red arrowheads in **Figures 3D,E,P,Q**) in the *Gli2^zfd/zfd^* mutants. At mamillary levels, however, the two lateral expression domains seem to have fused in a thickened midline [**Figures 3F,R**; this is also true of the expression of the truncated (inactive) form of *Gli2* in the mutant (**Figure 3L**)]. At this age, *Shh* expression is normally downregulated in the medial domain of the tuberal region (Manning et al., 2006; arrow in **Figure 5A**). In the *Gli2^zfd/zfd^* mutant this *Shh*-negative domain was absent (arrow in **Figure 5B**). *Nkx2-1,* a transcription factor gene defining regional specification of the basal hypothalamus (Kimura et al., 1996; Puelles et al., 2004, 2012), was expressed in an appropriate but smaller domain, with stronger expression shifted into the medial domain (**Figures 5C,D**). *Six3* is a transcription factor required for initiation of hypothalamic specification (Kobayashi et al., 2002). It is normally expressed strongly along the entire medial domain and flanking hypothalamus, except the mamillary part. *Six3* expression was severely reduced at both the infundibular (**Figures 5E,F**) and median eminence levels (**Figures 5G,H**). Together with the alterations in gene expression, we observed again a thickening of the medial domain of the tuberal region (arrowheads in **Figures 5F,H**). Analysis of *Six3* expression on sagittal sections at E12.5 (**Figures 5I,J**) confirmed *Six3* downregulation and a thickened medial domain of the *Gli2^zfd/zfd^* mutant (arrowheads in **Figures 5I,J**). Since expression of Six3 (**Figures 5E,F**) indicated alterations of the infundibulum, which is essential for pituitary development, we then examined the expression of appropriate gene markers for this region (**Figure 6**). Infundibular expression of *Tbx2* (Manning et al., 2006) and *Fgf8* (Ericson et al., 1998; **Figures 6A–D**), as well as expression of pituitary markers *Lhx3* (**Figures 6E,F**), and *Pitx2* (**Figures 6 G,H**) was completely lost in the *Gli2^zfd/zfd^* presumptive hypothalamus at E10.5 (see also Park et al., 2000). These results indicated that *Gli2* is required for appropriate development of the medial domain in the basal hypothalamus and for the development of the neurohypophysis.

### Arcuate, Ventromedial, and Mamillary Nuclei are Severely Reduced in Size in the *Gli2^**zfd/zfd**^* Mutant

**FIGURE 7:**
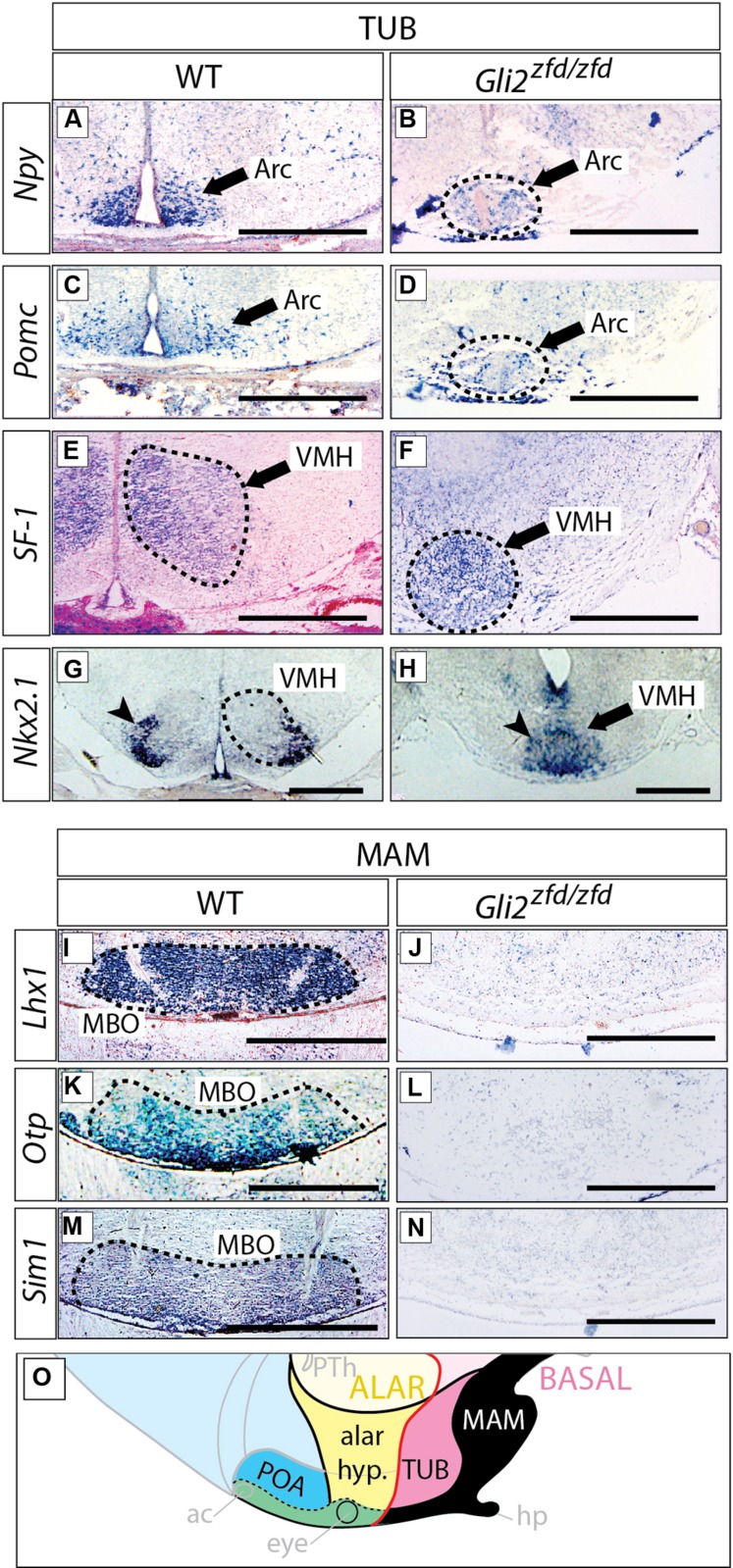
**Arcuate and ventromedial nuclei reduced, mamillary nucleus absent in the *Gli2^zfd/zfd^* mutant.** *In situ* detection of tuberal markers **(A–H)** and mamillary markers **(I–N)** on sections of E18.5 WT and *Gli2^zfd/zfd^* mouse brains as indicated. Arrowheads in **(G,H)** indicate the lateral portion of the ventromedial nucleus; Arc, arcuate nucleus; MBO, mamillary nucleus; VMH, ventromedial nucleus. Scale bars, 500 μm. **(O)** The proposed hypothalamic model (see **Figure 1B**) showing in black the area affected by the *Gli2* null mutation. Black arrows indicate the arcuate nucleus **(A–D)** or the ventromedial nucleus **(E,F,H)**. Dashed circles in **(B,D,E–G,I,K,M)** outline hypothalamic nuclei (as indicated).

We next analyzed the differentiation of the tuberal and mamillary regions in *Gli2^zfd/zfd^* brains at E18.5 (at this stage, characteristic neuronal nuclei are recognizable in the wildtype). *Npy*-expressing and *Pomc*-expressing neurons are specifically present in the arcuate nucleus (tuberal region; Elias et al., 1998; **Figures 7A,C**). In the *Gli2^zfd/zfd^* brain, the arcuate nuclei were not preserved as two distinct left and right domains. Instead, one single specifically labeled area was observed, unpaired and medial, sitting in the midline at the level of the tuberal area (dashed circle in **Figures 7B,D**). The third ventricle was abnormally absent at the site of this unpaired structure. Expression of *SF-1* (nuclear receptor *Nr5a1*) specifically labels the ventromedial nucleus of the hypothalamus (Ikeda et al., 1995; **Figure 7E**). In the *Gli2^zfd/zfd^* brain, *SF-1* was expressed in a median, unpaired group of cells (dashed circle in **Figure 7F**). The transcription factor *Nkx2-1* is specifically expressed in the lateral part of the wildtype ventromedial nucleus (Nakamura et al., 2001; **Figure 7G**), but formed one single medial domain in the *Gli2^zfd/zfd^* brain (**Figure 7H**). The transcription factor genes *Lhx1*, *Otp,* and *Sim1* are specifically expressed in the mamillary body (mamillary region) in the wildtype (Szabo et al., 2009a) but this expression was completely lost in the *Gli2^zfd/zfd^* mutant (**Figures 7I–N**). Together with the observations shown in **Figures 5** and **6**, these results indicate that *Gli2* is essential for the specification of the medial progenitor domain (Alvarez-Bolado et al., 2012) of the basal hypothalamus. The *Gli2^zfd/zfd^* mutant mice showed an altered latero-medial organization of the molecular pattern of the basal hypothalamus consistent with a loss of the medial markers (notably reduced *Six3* and loss of *Tbx2*, *Otp*, *Sim1,* and *Lhx1*) and derivatives (median eminence and neurohypophysis, mamillary body). The latter were substituted at the mutant midline by markers and derivatives typical of the lateral domain at this age, like *Nkx2.1, Npy, Pomc,* and* SF-1*. That the neurohypophysis is a derivative from this region has been described before (Pearson et al., 2011; Pearson and Placzek, 2013).

### *Gli2* in the Medial Progenitor Domain

In the early presumptive hypothalamus (E7.5 to E8.5), an unpaired medial progenitor domain (“med” in **Figures 2A** and **11A**) is specified, which gives rise to medially located nuclei like most of the arcuate nucleus, the medial portion of the ventromedial nucleus, the median eminence and the mamillary body (Alvarez-Bolado et al., 2012; derivatives of the acroterminal region, Puelles et al., 2012). This early domain and its lineage are strongly affected in the *Gli2^zfd/zfd^* mutant (**Figures 5**–**7**). *Gli2* expression overlaps with *Gli1* in the medial domain at E7.5 (Hui and Joyner, 1993). Since *Gli1* expression is diagnostic of Shh pathway activation, this indicates a Gli2A function. The strong *Gli2^zfd/zfd^* midline phenotype must be due to a requirement for *Gli2* expression in the medial domain at E7.5, since this domain does not show *Gli2* expression at later stages (**Figures 2** and **3**). Moreover, expression of *Gli1* and *Gli3,* normally absent from the midline at E8.5 (**Figures 2A,C**), is not ectopically upregulated in the *Gli2^zfd/zfd^* mutant (**Figures 2F,H**; i.e., no rescue). At E10.5, *Gli3,* and *Gli1* expression overlap in the midline (black arrowheads in **Figures 3A–C,M–O**) suggesting an activator role of Gli3 (Gli3A). However, both genes are strongly downregulated in the *Gli2^zfd/zfd^* midline at E10.5 (red arrowheads in **Figures 3D,E,P,Q**), again making a rescue of the *Gli2^zfd/zfd^* phenotype by a Gli3A function impossible.

### No Abnormal Phenotype in the *Gli3^**Xt-J/Xt-J**^* Basal Hypothalamus

**FIGURE 8:**
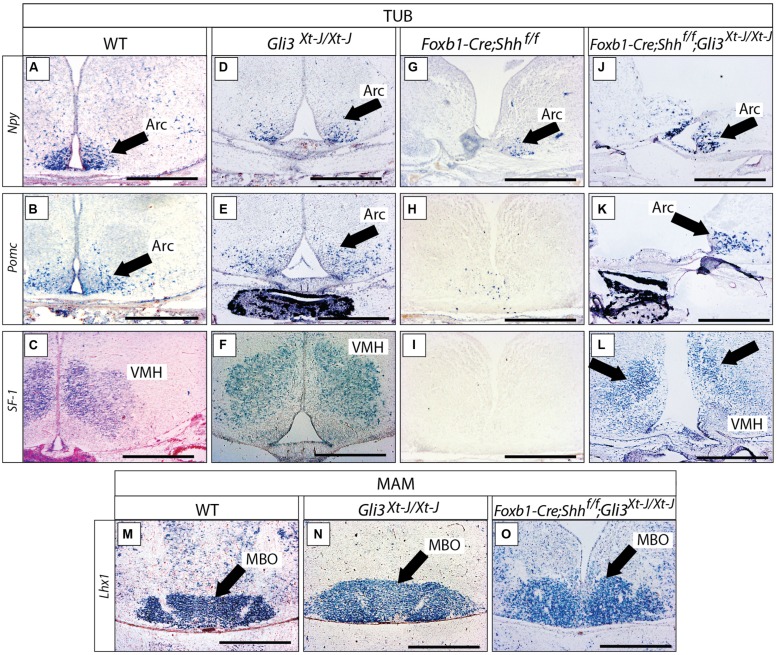
***Gli3* activator is dispensable for specification of the basal hypothalamus. (A–L)** *In situ* detection of tuberal marker genes on E18.5 mouse brain sections, markers and genotypes as indicated. Black arrows indicate the arcuate nucleus. Black arrows in L indicate the ventromedial nucleus. **(M–O)** *In situ* detection of mamillary marker gene *Lhx1* on E18.5 mouse brain sections, genotypes as indicated. Black arrows indicate the mamillary body (MBO). Arc, arcuate nucleus; MBO, mamillary body; VMH, ventromedial nucleus. Scale bars 500 μm.

We went on to analyze the developing *Gli3^Xt-J/Xt-J^* basal hypothalamus. At E18.5, expression of specific marker genes *Npy*, *Pomc* and *SF-1* in the tuberal region (**Figures 8A–F**) and of *Lhx1* in the mamillary region (**Figures 8M,N**), showed that a Gli3A function in presence of Gli2A is dispensable for the specification of the basal hypothalamus.

We next addressed the question of a possible Gli3R function in the developing hypothalamus. Gli3R function often results in negative regulation of tissue growth (by inducing cell death and reducing proliferation) and counteracting the ventralizing influence of Shh (Persson et al., 2002; Ruiz i Altaba et al., 2003). Therefore, loss of Gli3R could result in ventralization and/or an increased size of hypothalamic nuclei. Since Shh signaling counteracts the processing of Gli3 protein into its repressor form, experimental abolition of Shh signaling might result in overabundance of Gli3R. We investigated this possibility by analyzing mouse mutants lacking *Shh* expression in the neural tube (*Foxb1-Cre;Shh^f/f^* mutant, Szabo et al., 2009a). This mutant showed strong downregulation of three tuberal marker genes (*Npy*, *Pomc*, *SF-1*; **Figures 8G–I**) and had a more severe phenotype than the *Gli2^zfd/zfd^* mutants (**Figures 7B,D,F,H**; see also Szabo et al., 2009a; Shimogori et al., 2010). Given that Gli2A acts primarily at early stage of hypothalamic induction (see above) and following the logic of the Shh-Gli code (Bai et al., 2004), a possible explanation of this difference could be that, in the absence of neural *Shh*, formation of Gli3R is not prevented. A prediction of this hypothesis is that, in the absence of both neural* Shh* and *Gli3* the tuberal region would have a less marked phenotype. We tested this prediction by analyzing mutants deficient not only in neural *Shh* but also in *Gli3* (*Foxb1-Cre;Shh^f/f^;Gli3^Xt-J/Xt-J^* mutants). These showed essentially correct marker expression in the arcuate and ventromedial nuclei (although the expression domains appeared somewhat reduced and distorted; **Figures 8J–L**) suggesting that the phenotype in *Foxb1-Cre;Shh^f/f^* mutants is at least partially caused by upregulated Gli3R activity. The same reasoning applies to the mamillary body (mamillary region), which is extremely reduced in the *Foxb1-Cre;Shh^f/f^* mutant (Szabo et al., 2009a) but appears normal in the *Gli3^Xt-J/Xt-J^* mutant and in *Foxb1-Cre;Shh^f/f^;Gli3^Xt-J/Xt-J^* double mutants (**Figures 8M–O**). We conclude that *Gli3* is dispensable for overall hypothalamic specification. Moreover, it is likely that the upregulation of Gli3R is the main contributor to the defects in the tuberal and mamillary hypothalamus when neural *Shh* is inactivated, which would be consistent with the classical Shh-Gli code in the spinal cord (Bai et al., 2004).

### A Possible *Gli3* Activator Function in the Lateral Hypothalamic Area of the *Gli2^**zfd/zfd**^* Mutant

**FIGURE 9:**
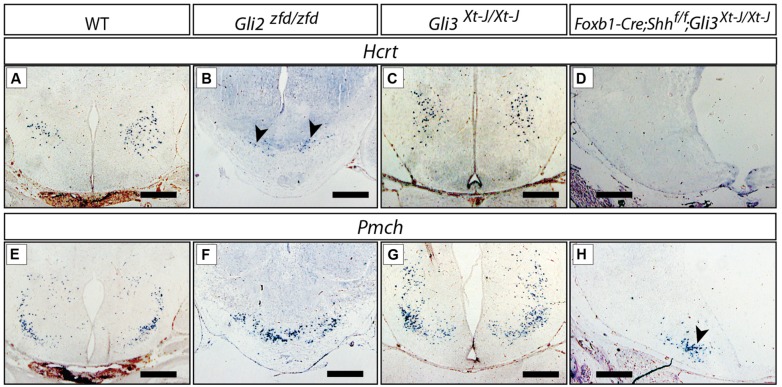
***Gli* mutant phenotypes in the LHA.** RNA *in situ* detection of lateral hypothalamic markers *Hcrt/Orexin* and *Pmch* on E18.5 mouse brain sections, genotypes as indicated. Arrowheads in **(B,H)**, point at smaller groups of cells. Scale bars, 500 μm.

The LHA is a large and morphologically complex region and with key functions in the regulation of behavioral state and arousal mechanisms [reviewed in Swanson (2000)]. Analysis of *Foxb1-Cre;Shh^f/f^* mutants has shown that expression of *Shh* by the forebrain is essential for its specification (Szabo et al., 2009a). *Hypocretin/orexin (Hcrt;* Hungs and Mignot, 2001; **Figures 9A–D**) and *pro-melanin-concentrating hormone (Pmch;* Croizier et al., 2013; **Figures 9E–H**), essential modulators of metabolism and behavior, are among the very few specific marker genes of restricted groups of LHA neurons. The *Gli3^Xt-J/Xt-J^* brain did not show changes in *Hcrt* (**Figure 9C**) or *Pmch* expression (**Figure 9G**), indicating that Gli3A is normally not involved in the specification of the LHA. In the *Gli2^zfd/zfd^* mutant mice, only a few scattered *Hcrt*-expressing cells were present, and they were displaced toward the midline from their normal lateral position (arrowheads in **Figure 9B**). The number of *Pmch*-expressing neurons in the *Gli2^zfd/zfd^* mutant seemed not altered, but the cells tended to gather in the midline, similar to *Hcrt* cells (**Figure 9F**). This indicates that *Gli2* is dispensable for the generation of *Pmch*-expressing cells, their altered position being rather a phenotypic consequence of the missing medial domain in this mutant (**Figures 5** and **6**). The phenotype of *Foxb1-Cre;Shh^f/f^* mutants in this area (*Hcrt* cells are absent, and *Pmch* cells severely reduced Szabo et al., 2009a) is stronger that that of *Gli2^zfd/zfd^* mutants. We went on to address the possibility that a compensatory Gli3A function could explain the relatively mild LHA phenotype of *Gli2^zfd/zfd^* mutant mice. To test this hypothesis, we examined double mutants deficient in neural *Shh* and *Gli3 (Foxb1-Cre;Shh^f/f^;Gli3^Xt-J/Xt-J^*) and found a phenotype similar to that of the *Foxb1-Cre;Shh^f/f^* mutants (*Hcrt* cells absent, *Pmch* cells severely reduced; **Figures 9D,H**), but more pronounced than that of *Gli2^zfd/zfd^* mutants (**Figures 9B,F**). This indicates that, in the LHA of the *Gli2^zfd/zfd^* brain, Gli3A might compensate for the loss of Gli2A. This would be consistent with *Gli1* still being expressed in the lateral domain of *Gli2^zfd/zfd^* mutants at E8.5 (**Figure 2E**).

We concluded that, for the specification of the LHA progenitors within the lateral progenitor domain, a Gli2A function is needed which can be partially substituted for by Gli3A.

### *Gli3* in Mamillary Neurogenesis

Differences in size can be due to quantitative changes in precursor generation (symmetric cell divisions) at an early stage or to later changes in neuron generation (asymmetric cell divisions). Shh is essential for the expansion of neural precursors in the early development of this region (Rowitch et al., 1999; Ishibashi and McMahon, 2002).

Here, we wanted to address the contribution of Shh-Gli to the neurogenesis of hypothalamic nuclei.

**FIGURE 10:**
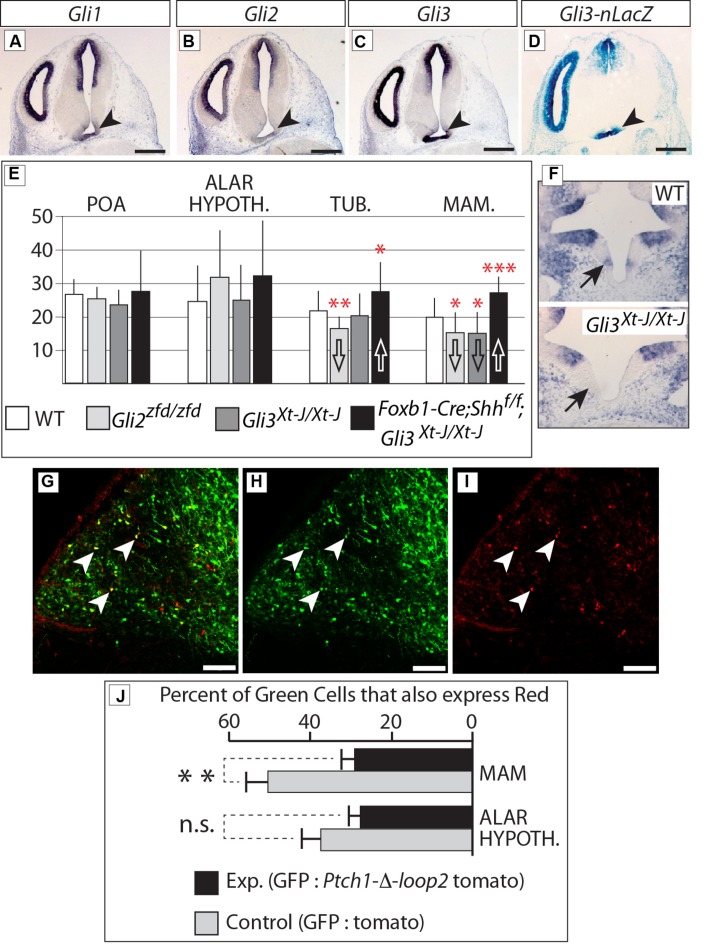
***Gli3* promotes proliferation in the mamillary region. (A–C)** *In situ* detection of *Gli* genes on E12.5 WT mouse brain sections. Arrowheads indicate the mamillary region. **(D)** LacZ reporter detection on an E12.5 *Gli3-nlacZ* knock-in mouse brain section; arrowhead indicates the mamillary region. **(E)** BrdU-labeled cells per bin at E12.5, genotypes as indicated. Unpaired *t*-test, two-tailed, mean ± SD; ^∗^*p* ≤ 0.05, ^∗∗^*p* ≤ 0.01, ^∗∗∗^*p* ≤ 0.001. **(F)** *Gli1* expression in the medial domain of the hypothalamus (arrows) on E10.5 horizontal sections of WT (upper panel) and* Gli3^Xt-J/Xt-J^* (lower panel) embryos. **(G–I)** Labeled cells in the mamillary region of WT E18.5 embryos after *in utero* electroporation with GFP and Ptch-δ-loop-tomato DNA constructs at E12.5. White arrowheads show double-labeled cells. **(J)** Percent of GFP-expressing cells co-expressing red reporter “tomato” after *in utero* electroporation of control (white bars) or experimental (black bars) constructs, in two different regions, as indicated. Unpaired *t*-test, two-tailed, mean ± SD; ^∗∗^*p* ≤ 0.01; n.s., non-significant.

*In situ* analysis of *Gli* family expression at E12.5 (**Figures 10A–C**) showed *Gli1* and *Gli3* (arrowheads in **Figures 10A,C**) expression in the mamillary region. *Gli2* expression on the contrary was very low or absent (arrowhead in **Figure 10B**). In agreement, a *Gli3* reporter mouse line (*Gli3-nLacZ* knock-in) showed strong beta-galactosidase labeling in the mamillary region (arrowhead in **Figure 10D**).

Therefore, we labeled proliferating cells in the neural tube by injecting BrdU in pregnant mice at E12.5 [i.e., at the peak of neurogenesis in the mouse hypothalamus (Ishii and Bouret, 2012)] and collecting the embryonic brains 3 h later. Our results (**Figure 10E**) show that proliferation during the neurogenic period in the mamillary region was reduced in the *Gli3^Xt-J/Xt-J^* mutant. *Gli1* expression is diagnostic of *Shh* pathway activation, and can be found in the mamillary region at E10.5 (**Figure 10F**, upper panel), but it is lost in the *Gli3^Xt-J/Xt-J^* mutant (**Figure 10F**, lower panel), indicating failure of this domain to activate the *Shh* pathway in the mutant, in agreement with the reduced proliferation. BrdU labeling in this region was also reduced in the *Gli2^zfd/zfd^* mutant (**Figure 10E**), which we interpret as a consequence of the defect in midline development in these mutants (**Figures 5** and **6**). Intriguingly, deficiency in both neural *Shh* and *Gli3* increased neurogenesis, particularly in the mamillary region (**Figure 10E**; see Discussion).

We then approached this issue experimentally by specifically blocking the Shh pathway in the hypothalamus of mouse embryos developing *in utero*. We electroporated wildtype embryos at E12.5 (**Figures 10G–I**) with *EGFP*-expressing reporter constructs mixed with constructs expressing the loss-of-function Shh receptor *Ptch1-δ-loop2* plus the red fluorescent reporter *tdTomato* [similar to the one used by Briscoe et al. (2001); see Materials and Methods]. The results show that cells expressing high levels of *Ptch1-delta-loop2* plus* tdTomato* in the mamillary region are less proliferative, while the same experiment did not alter neurogenesis in the alar hypothalamus (**Figure 10J**). We concluded that proliferation during the neurogenic period in the alar portion of the hypothalamus is not directly affected by Shh-Gli, while *Gli3* has a role as an activator inducing neurogenesis at later stages in the basal regions.

## Discussion

**FIGURE 11:**
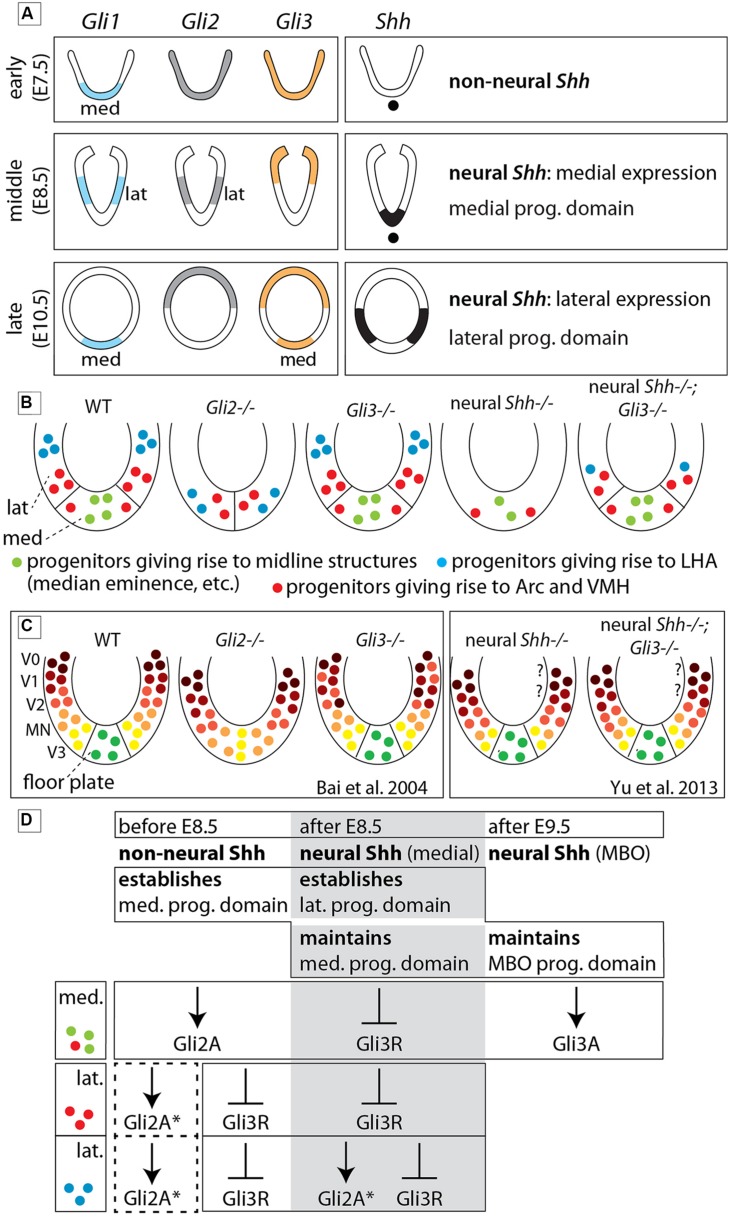
**The role of each Gli protein in the basal hypothalamus of the mouse. (A)** Diagram showing expression domains of the Gli factors and *Shh* in the presumptive hypothalamus at the early, middle, and late phases. “lat” and “med,” lateral and medial domains, respectively (early phase according to Hui et al., 1994). **(B)** Summary diagrams of progenitor domains (neuroepithelium) of the basal hypothalamus in WT and mutants as deduced from phenotype analysis in the present study. **(C)** Diagrams comparable to those in **(B)** representing the progenitor domains in the spinal cord of WT and mutants as reported in the literature (as indicated). Question marks indicate that the V0/V1 domains have not been investigated. **(D)** Specific contribution of Gli proteins to the specification of the medial and lateral progenitor domains in three successive stages of development. Dotted square, possible influence of Gli2A on lateral progenitors before E8.5. The asterisk (^∗^) means that loss of GliA2 could be compensated by Gli3A. MBO, mamillary body. See Discussion for details.

We asked which combinations of GliA and GliR specify different hypothalamic regions, and which members of the Gli family perform GliA or GliR functions. Therefore we interpreted mutant phenotypes with the help of Shh and Gli expression patterns (**Figure 11A**), hypothalamic progenitor domains (Alvarez-Bolado et al., 2012; **Figure 11B**), the Shh-Gli code established for the spinal cord (Bai et al., 2004) and a hypothalamic model (**Figure 1B**; Puelles et al., 2012). We uncovered strong differences in *Gli* gene requirements between alar and basal hypothalamus as predicted by the model. Null mutations of *Gli2* or *Gli3* do not alter the overall specification of the alar portions, including preoptic (actually telencephalic) and alar hypothalamus (or “anterior region”). In the basal regions (tuberal and mamillary), however, *Gli2* is indispensable for the development of the medial progenitor domain and its derivatives but it is partly dispensable for the lateral progenitor domain (**Figures 11B,D**). *Gli3* is dispensable for overall specification of the wildtype hypothalamus, but Gli3A has a late influence on mamillary proliferation. Finally, medial progenitor domain specification is dependent on Shh of non-neural source (prechordal plate, notochord), while the lateral progenitor domain is strongly dependent on neural Shh, in whose absence ectopic upregulation of Gli3R causes a severe phenotype. In this way, the notorious anatomical complexity of the hypothalamus depends on combinations of specification timing, progenitor domain, Shh source, *Gli* gene dependence, and alar vs. basal position (**Figure 11D**).

### Gli2A, Gli3A, and repression of Gli3R in hypothalamus specification

*Gli2^zfd/zfd^* mutants lack a floor plate and its flanking cells from spinal cord to midbrain (Matise et al., 1998; Park et al., 2000). We extend this result to the rostral end of the floor plate (the mamillary region Puelles et al., 2012), and beyond this point, through the medial progenitor domain of the entire basal hypothalamus. Therefore, in the *Gli2^zfd/zfd^* mutant, the median eminence, pituitary and mamillary body are missing as well as part of the arcuate and ventromedial nuclei (**Figure 11B**).

In spinal cord, *Gli3* has important repressor (Gli3R; Litingtung and Chiang, 2000; Persson et al., 2002) and, in the absence of Gli2A, activator (Gli3A) functions (Bai et al., 2004). The lack of a pronounced phenotype in the *Gli3^Xt-J/Xt-J^* mutant hypothalamus indicates that under normal conditions either Gli3A is not important or that Gli2A can substitute for Gli3A. However, in the *Gli2^zfd/zfd^* mutant, the lateral progenitor domain is still able to produce part of the ventromedial and the arcuate nuclei and also the LHA (with only minor alteration; **Figures 9B,F**) – this can either be explained through rescue by a Gli3A function or it indicates that GliA is not essential for the induction of the lateral progenitor domain. A partial compensation through Gli3A (asterisk in **Figure 11D**) is supported by *Gli1* expression in the lateral progenitor domain of the *Gli2^zfd/zfd^* mutants (**Figure 2F**), where it partially overlaps with *Gli3* (**Figure 2H**). The suppression of Gli3R function by non-neural and neural Shh appears to be essential for the development of the hypothalamus (**Figure 11D**), since the phenotype of the ventral forebrain in *Shh* null mutants (Chiang et al., 1996) is more severe than in *Gli2^zfd/zfd^* mutants and the phenotype of the lateral progenitor domain in *Foxb1-Cre;Shh^f/f^* mutants (Szabo et al., 2009a) is more severe than in *Gli2^zfd/zfd^* mutants. In addition, the medial and lateral progenitor domains of *Foxb1-Cre;Shh^f/f^* mutants are largely rescued in *Foxb1-Cre;Shh^f/f^;Gli3^Xt-J/Xt-J^* mice (**Figures 8G–I,J–L**). Finally, *Hcrt*+ neurons (part of the LHA) are reduced in *Gli2^zfd/zfd^* mutants and completely lost in *Foxb1-Cre;Shh^f/f^* mutants and even in *Foxb1-Cre;Shh^f/f^;Gli3^Xt-J/Xt-J^* brains, suggesting that their progenitors are uniquely specified by GliA or need GliA later or for a longer time than other progenitors in the lateral domain (**Figure 11D**). This is consistent with the very restricted place and time of neurogenesis of *Hcrt*+ neurons (Amiot et al., 2005). As mentioned in Section “Materials and Methods,” we have not been able to obtain double mutant *Gli2-Gli3* embryos.

As for the alar hypothalamus and preoptic area, they are strongly dependent on Shh for their development, while *Gli2^zfd/zfd^* or *Gli3^Xt-J/Xt-J^* show no – or only subtle – phenotypes in these areas. This indicates that either Gli2A and Gli3A can fully substitute for each other in these regions, or they depend on suppression of Gli3R rather than induction of GliA by *Shh* for their specification (Chiang et al., 1996; Rallu et al., 2002).

### The Hypothalamic Version of the Shh-Gli Code

In the spinal cord (**Figure 11C**), signaling by notochordal Shh is sufficient to generate the proper pattern of ventral progenitor gene expression (Matise et al., 1998; Jeong and McMahon, 2005), whereas ongoing Shh signaling from the floor plate (neural *Shh*) is necessary to maintain progenitor domain formation during neurogenesis (Dessaud et al., 2010) and for oligodendrocyte specification (Yu et al., 2013). We show that Shh of non-neural origin specifies the medial progenitor domain through Gli2A at an early stage, while neural Shh specifies the lateral progenitor domain at a later stage, probably by counteracting ectopic GliR function and, in the case of LHA progenitors, by inducing Gli2A (see above).

In both spinal cord (Bai et al., 2004; **Figure 11C**) and hypothalamus (**Figures 11B,D**), *Gli2* performs the main GliA function. *Gli2,* however, is required for the induction of *Shh* expression in the floor plate (Matise et al., 1998), but not in the hypothalamus (**Figure 2I**).

Opposite gradients of GliA and GliR underlie the precise dorsoventral polarity of the spinal cord (Litingtung and Chiang, 2000; Persson et al., 2002) and hypothalamic specification requires counteracting Gli3R by Shh. Additionally, in the chicken hypothalamus, Gli3R activity is involved in *Pax7* de-repression in some progenitors (Ohyama et al., 2008).

Finally, a Gli3A function is required for mamillary proliferation during the neurogenic phase (**Figure 10E**). The mamillary region overgrowth in *Foxb1-Cre;Shh^f/f^;Gli3^Xt-J/Xt-J^* mutants (**Figure 10E**) parallels the abnormally increased size of the spinal cord in *Gli2^zfd/zfd^;Gli3^Xt-J/Xt-J^* mutants. It remains unclear why inactivation of all Shh signaling results in a proliferation increase (Bai et al., 2004).

### Acroterminal Region vs. Floor Plate

We have mapped the *Gli2^zfd/zfd^* phenotype (**Figure 7O**) on a genetic-molecular model of the developing hypothalamus (Puelles et al., 2012) in which the ventral and dorsal midlines do not meet at a hypothetical “tip” of the neural tube (**Figure 1B**). Rather, the model proposes that the rostral end of the tube is closed by a “lid” in the form of a transverse structure called acroterminal region, which does not share the typical characteristics of the floor plate—e.g., it does not express *Foxa2* (Ruiz i Altaba et al., 1995; Dale et al., 1999), undergoes complex, specific regulation (Ohyama et al., 2005; Manning et al., 2006; Ohyama et al., 2008; Trowe et al., 2013) and, as we show here, it is strongly neurogenic, not a property of the floor plate (except in the midbrain Kittappa et al., 2007; Ono et al., 2007; Bonilla et al., 2008). The histologically recognizable floor plate expresses *Shh*, *Ntn1*, *Lmxb1*, *Foxa1*, and *Nr4a2* (Allen-Institute-for-Brain-Science, 2009; Puelles et al., 2012), is induced by the underlying notochord, and it ends rostrally at mammillary level (Puelles et al., 2012). Beyond mammillary level, the acroterminal region extends all the way through the tuberal region, alar hypothalamus and preoptic region and up to the anterior commissure, it is transversally oriented (has alar and basal portions) and strongly patterned (probably by the underlying prechordal plate) and generates, among other, the median eminence, infundibulum, neurophypophysis, and eyes.

The dorso-ventral and rostro-caudal axes of the embryonic neural tube, considered in this way, are at a 90° angle with those of the adult brain as they are usually considered; i.e., the adult rostro-caudal axis would be the dorso-ventral axis in our model. If this discrepancy will eventually be corrected remains open. The connectivity and function of the classical regions of the hypothalamus and the behavioral control column (Swanson, 2000) are not otherwise challenged by the proposed nomenclature (Puelles et al., 2012).

### The *Gli2^**zfd/zfd**^* Phenotype and the Hypothalamic Model

The *Gli2^zfd/zfd^* hypothalamic phenotype can be cleanly mapped (**Figure 7O**) on the model of the embryonic hypothalamus (Puelles et al., 2012), which in turn receives experimental confirmation from our work. The basal regions depend specifically on *Gli2*. The alar hypothalamus and preoptic region, on the contrary, are not strictly dependent on Gli2A or Gli3A and are therefore genetically different. In this way, the basal/alar boundary, one main insight of the model, is confirmed. The basal part has unique genetic requirements, as much in the floor plate as in the acroterminal region, which are difficult to reconcile with a conventional rostral-caudal hypothalamic orientation (**Figure 1A**). Moreover, the medial and lateral progenitor domains of the basal hypothalamus (Alvarez-Bolado et al., 2012) can be mapped on the model too, corresponding to acroterminal and terminal hypothalamus (Puelles et al., 2012). Mapping other mutant phenotypes will refine the model and reveal fundamental aspects of brain development and organization. The patterning of the acroterminal region by the prechordal plate, for instance, is an open question.

## Conflict of Interest Statement

The authors declare that the research was conducted in the absence of any commercial or financial relationships that could be construed as a potential conflict of interest.
