# Corrigendum: Differential requirements for *Gli2* and *Gli3* in the regional specification of the mouse hypothalamus

**DOI:** 10.3389/fnana.2015.00058

**Published:** 2015-05-13

**Authors:** Roberta Haddad-Tóvolli, Fabian A. Paul, Yuanfeng Zhang, Xunlei Zhou, Thomas Theil, Luis Puelles, Sandra Blaess, Gonzalo Alvarez-Bolado

**Affiliations:** ^1^Department of Neuroanatomy, University of Heidelberg Heidelberg, Germany; ^2^Laboratory of Neurodevelopmental Genetics, Life and Brain Center, Institute of Reconstructive Neurobiology, University of Bonn Bonn, Germany; ^3^Centre for Integrative Physiology, University of Edinburgh Edinburgh, UK; ^4^Department of Morphology, University of Murcia and IMIB, Murcia Murcia, Spain

**Keywords:** embryo, *Gli1*, *Gli2*, *Gli3*, hypothalamus, mouse, mutant, Shh

By mistake, Figure 2 of the article by Haddad-Tóvolli et al. (2015) showed in panels **(A)** and **(B)** the same image of *Gli1* expression in E8.5 wildtype mouse embryos. It should have shown *Gli1* expression in (**A**) and *Gli2* expression in (**B**). Therefore, we provide a corrected Figure 2, now with panel (**B**) showing *Gli2* expression, as we originally intended and as the Figure legend indicates. This is a minor change not affecting the scientific content of the article.

**Figure 2:**
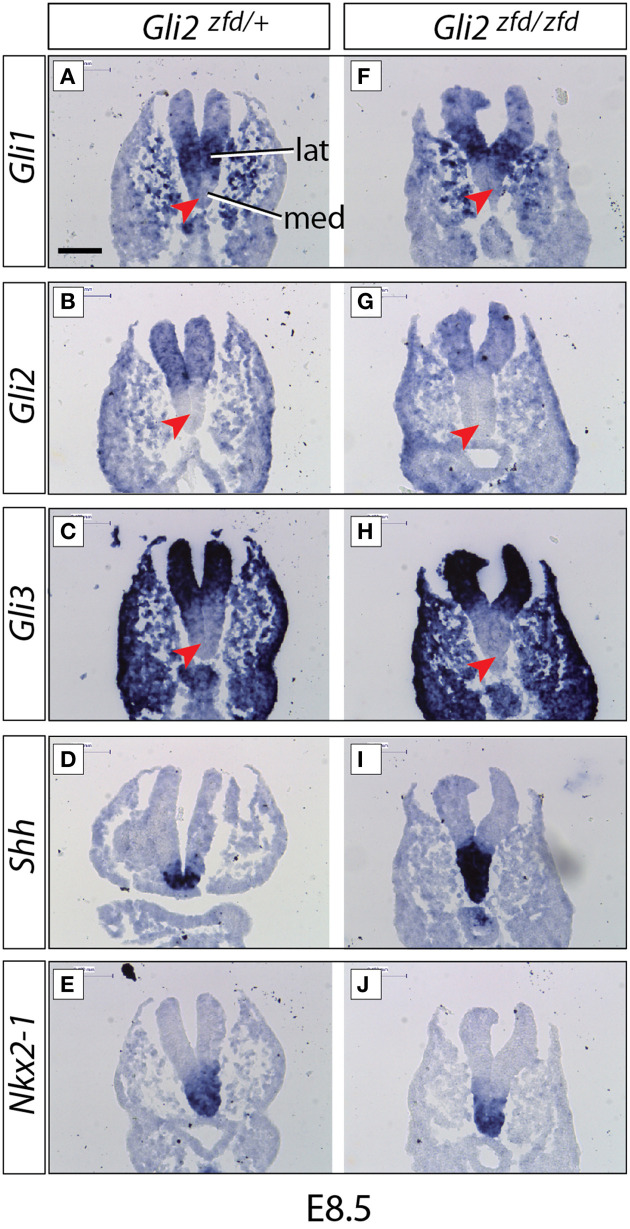
**Expression of *Gli* genes in the presumptive hypothalamus at E8.5**. *In situ* detection of marker gene expression in *Gli2^zfd/+^* and *Gli2^zfd/zfd^* mutant E8.5 embryos as indicated. “lat” and “med” in **(A)** indicate progenitor domains. Red arrowheads in **(A–C)** and **(F–H)** indicate lack of expression in the medial progenitor domain. *Nkx2-1* expression **(E,J)** identifies the presumptive hypothalamus. Scale bar (in **A**) 100 μm.

## Conflict of interest statement

The authors declare that the research was conducted in the absence of any commercial or financial relationships that could be construed as a potential conflict of interest.

